# Qualitätsverbessernde Maßnahmen in der Versorgung von kritisch kranken Intensivpatienten mit Nierenersatztherapie bei akuter Nierenschädigung: Positionspapier der Sektion Niere der DIVI unter Mitarbeit von DGAI und DGIIN

**DOI:** 10.1007/s00063-020-00734-8

**Published:** 2020-10-06

**Authors:** Detlef Kindgen-Milles, Peter Heering, Melanie Meersch-Dini, Michael Schmitz, Michael Oppert, Stefan John, Achim Jörres, Alexander Zarbock, Carsten Willam

**Affiliations:** 1 Klinik für Anästhesiologie, Universitätsklinikum Düsseldorf, Moorenstr. 5, 40225 Düsseldorf, Deutschland; 2 Klinik f. Nephrologie und Allgemeine Innere Medizin, Städtisches Klinikum Solingen, Gotenstraße 1, 42653 Solingen, Deutschland; 3 Klinik für Anästhesiologie, operative Intensivmedizin und Schmerztherapie, Universitätsklinikum Münster, Albert-Schweitzer-Campus 1, Gebäude A1, 48149 Münster, Deutschland; 4 Zentrum für Notfall- und Intensivmedizin, Klinikum Ernst von Bergmann, Charlottenstr. 72, 14467 Potsdam, Deutschland; 5 Universitätsklinik Medizinische Klinik 8 – Kardiologie, Paracelsus Medizinische Privatuniversität Nürnberg, Breslauer Straße 201, 90471 Nürnberg, Deutschland; 6 Klinik für Nephrologie, Transplantationsmedizin und internistische Intensivmedizin, Medizinische Klinik I Merheim, Ostmerheimerstr. 200, 51109 Köln, Deutschland; 7 Medizinische Klinik 4, Universität Erlangen, Ulmenweg 18, 91054 Erlangen, Deutschland

**Keywords:** Akute Nierenschädigung, Nierenersatztherapie, Dialyse, Hämofiltration, Qualitätsstandards, Acute renal failure, Renal replacement therapy, Dialysis, Hemofiltration, Quality standards

## Abstract

Die Nierenersatztherapie ist neben der Beatmung eines der wichtigsten und am häufigsten angewendeten Organersatzverfahren in der täglichen Praxis in der Intensivmedizin. Im Gegensatz zur Beatmungstherapie sind Qualitätsstandards für die Nierenersatztherapie weniger gut definiert und bekannt. In diesem Positionspapier der Deutschen Interdisziplinären Vereinigung für Intensivmedizin (DIVI) beschreiben wir Qualitätsstandards zur Nierenersatztherapie mit dem Ziel die Behandlungsqualität der Patienten mit einem schweren akuten Nierenversagen zu verbessern.

## Abstract (en)

Renal replacement therapy is after mechanical ventilation one of the most important and frequently used organ replacement therapies in daily routine intensive care practice. In contrast to mechanical ventilation, quality standards for renal replacement therapy are less well known and defined. In this position paper of the German Interdisciplinary Association for Intensive Care and Emergency Medicine, we describe quality standards of renal replacement procedures in order to improve therapy of patients with severe acute kidney injury.

## Einführung

Eine akute Nierenschädigung („acute kidney injury“; AKI) gemäß der KDIGO-Klassifikation von 2012 [1] betrifft nach aktuellen epidemiologischen Daten etwa 50 % aller Intensivpatienten. Etwa 15 % aller Intensivpatienten müssen während ihrer Behandlung auf der Intensivstation einer Nierenersatztherapie unterzogen werden [2]. Bei den betroffenen Patienten ist die Liegedauer auf der Intensivstation und im Krankenhaus verlängert und die Gesamtbehandlungskosten steigen. Die Hauptursache für eine AKI in der Intensivmedizin ist mit etwa 40 % eine Sepsis, gefolgt von Volumenmangelzuständen (34 %), einer Medikamententoxizität (14 %) sowie einem kardialen Pumpversagen (13 %; [2]). Klassische Nierenerkrankungen, die außerhalb der Intensivmedizin häufig zu akuten Nierenfunktionsstörungen führen können, sind in der Intensivmedizin eher selten. Die Letalität kann insbesondere in der häufig auftretenden Kombination mit einem septischen Schock 50 % übersteigen [3].

Neben der hohen Akutletalität entwickelt sich bei 10–25 % der Patienten mit AKI II–III in den nächsten 10 Jahren eine chronische Nierenerkrankung bis hin zu einer terminalen Niereninsuffizienz (HR 4,8), darüber hinaus ist auch im Langzeitverlauf die Sterblichkeit signifikant erhöht (HR 1,8; [4]).

Aus diesen Gründen sollten Risikopatienten frühzeitig identifiziert, adäquate Präventionsmaßnahmen eingeleitet und aggravierende Faktoren konsequent eliminiert werden. Beispielsweise sollte vor invasiven Prozeduren in der Kardiologie oder Kardiochirurgie bzw. per se vor großen operativen Eingriffen eine entsprechende Optimierung des Behandlungspfads erfolgen [5].

Auf der Intensivstation müssen diese Maßnahmen konsequent fortgesetzt werden. Eine renale Protektion ist somit integraler Bestandteil des in der Regel komplexen Gesamtbehandlungskonzepts [6]. Eine Nierenersatztherapie kann schließlich erforderlich werden, um lebensbedrohliche Störungen, wie eine Überwässerung, Elektrolytstörungen und Störungen des Säure-Basen-Haushalts, zu behandeln sowie die Entstehung einer Urämie zu verhindern.

Die Durchführung einer Nierenersatztherapie ist eine aufwändige und invasive Prozedur in einem komplexen Behandlungsumfeld. In Anbetracht der großen Zahl betroffener Patienten ist sie eine der Kernaufgaben moderner Intensivmedizin. Neben einer Nierenersatztherapie sind regelhaft medikamentöse und/oder weitere apparative Verfahren zur Unterstützung anderer Organsysteme notwendig, da eine AKI auf der Intensivstation meist Bestandteil eines Mehrorganversagens ist. Die Nierenersatztherapie muss deshalb in das ganzheitliche Behandlungskonzept für den Intensivpatienten integriert werden, insbesondere muss die Nierenersatztherapie kompatibel zu anderen apparativen Organunterstützungssystemen angewandt werden. Dazu müssen strukturelle, personelle und prozedurale Voraussetzungen geschaffen werden. Die hier vorgeschlagenen Maßnahmen sollen Mindestvoraussetzungen für die sichere Durchführung einer Nierenersatztherapie bei Intensivpatienten mit AKI beschreiben.

Die Behandlung dieser Patienten beschränkt sich aber nicht nur auf den unmittelbaren Zeitraum der AKI. Vielmehr ist eine kontinuierliche Nachbehandlung erforderlich – bei vielen Patienten über Jahre hinaus, um frühzeitig die Entwicklung einer chronischen Nierenerkrankung zu erkennen und um dann entsprechende präventive Maßnahmen rechtzeitig einleiten zu können. Entsprechend aktueller Empfehlungen sollten in Deutschland unter Beteiligung der in der Behandlungskette involvierten Fachdisziplinen schnell Strukturen geschaffen werden, um eine adäquate kontinuierliche Nachsorge unter besonderer Berücksichtigung der Entwicklung einer chronischen Nierenerkrankung zu gewährleisten. Eine solche Nachsorge kann nach Datenlage die Morbidität, Letalität und möglicherweise auch die Lebensqualität im Langzeitverlauf günstig beeinflussen [7–9].

## Strukturvoraussetzungen

Die Sektion Niere der Deutschen Interdisziplinären Vereinigung für Intensivmedizin (DIVI) schlägt unter Berücksichtigung der spezifischen Versorgungsstruktur in Deutschland folgende Strukturvoraussetzungen für die Durchführung einer Nierenersatztherapie auf der Intensivstation vor.

- Die Strukturvoraussetzung für die Durchführung mindestens der einfachen intensivmedizinischen Komplexbehandlung liegt vor.
- Mindestens eine Form der Nierenersatztherapie ist 24/7 verfügbar.
- Ein nephrologisches Konsil sollte innerhalb von 48 h durchführbar sein.
- Eine qualifizierte abdominelle Sonographie der Niere, der ableitenden Harnwege sowie der unteren Hohlvene ist kurzfristig verfügbar.
- Es findet eine regelmäßige interprofessionelle Teamschulung zum Thema AKI und Nierenersatztherapie statt, an der jeder Mitarbeiter mindestens einmal jährlich teilzunehmen hat.
- Ein zertifiziertes klinisch-chemisches Labor sowie die Möglichkeit zur Urindiagnostik einschließlich Urinmikroskopie sind vorhanden.
- Es besteht eine Kooperation mit einer urologischen Fachabteilung zur Organisation urologischer Notfalleingriffe.

## Prozessvoraussetzungen

Die Sektion Niere der DIVI schlägt unter Berücksichtigung der spezifischen Versorgungsstruktur in Deutschland folgende Prozessvoraussetzungen für die Durchführung einer Nierenersatztherapie auf der Intensivstation vor.

- Ein Behandlungsbeginn muss unverzüglich nach Indikationsstellung möglich sein.
- Die Indikationsstellung für den Beginn der akuten Nierenersatztherapie erfolgt in der Regel durch einen Facharzt mit Zusatzweiterbildung Intensivmedizin oder einen Facharzt für Nephrologie,
- Im Notfall (etwa bei Vorliegen lebensbedrohlicher Veränderungen des Wasser‑, Elektrolyt- und Säure-Basen-Haushalts; KDIGO Leitlinie 5.1.1) darf hiervon abgewichen werden, jedoch muss die Indikation innerhalb von 24 h nach Behandlungsbeginn mit einem Nierenersatzverfahren durch einen Facharzt mit Zusatzweiterbildung Intensivmedizin oder einen Nephrologen bestätigt werden.
- Eine der Erkrankungsschwere angemessene invasive bzw. nichtinvasive hämodynamische Überwachung ist sicherzustellen.
- Es muss eine schriftliche Verfahrensanweisung „Optimierung der Behandlung vor Beginn einer Nierenersatztherapie“ vorhanden sein. Diese muss mindestens folgende Abschnitte umfassen:
  - Optimierung von Volumenstatus und Hämodynamik;
  - Überprüfung der Medikation;
  - diagnostische Maßnahmen;
  - Überwachungsmaßnahmen.
- Es muss eine schriftliche Verfahrensanweisung „Durchführung einer Nierenersatztherapie“ vorhanden sein. Diese muss mindestens folgende Abschnitte umfassen:
  - Indikation und Behandlungsbeginn;
  - vaskulärer Zugang;
  - Verfahrenswahl;
  - Nierenersatztherapiedosis (NET-Dosis);
  - Antikoagulationsverfahren:
    - Standardkonzept;
    - Konzept für blutungsgefährdete oder blutende Patienten, z. B. regionale Zitratantikoagulation;
    - Konzept für Patienten mit HIT-Typ 2;
  - Ernährung unter Nierenersatztherapie;
  - Anpassung der Medikation unter Nierenersatztherapie;
  - Behandlungsende;
  - Überleitungsmanagement und Nachkontrolle.
- Die Dokumentation der Behandlung muss in Form eines täglichen Dialyseprotokolls mindestens folgende Parameter erfassen:
  - Behandlungsdauer, Verfahren, verwendeter Filter;
  - Grund für eine Unterbrechung;
  - Blutfluss;
  - verschriebene und applizierte NET-Dosis;
  - Festlegung des Bilanzziels und der Ultrafiltrationsmenge unter Berücksichtigung der individuellen hämodynamischen Situation;
  - Antikoagulation;
  - Körpertemperatur;
  - Komplikationen.
- Folgende Laborparameter sind bei jedem Patienten mindestens einmal täglich zu erheben:
  - Natrium, Chlorid, Kalium, ionisiertes und Gesamtkalzium, Phosphat, Magnesium (insbesondere bei Zitratverfahren);
  - pH, Bikarbonat, Base Excess;
  - Laktat;
  - Harnstoff und Kreatinin;
  - Gerinnungsstatus;
  - Blutzucker;
  - Serumalbumin und Gesamtprotein mindestens einmal zu Beginn der Behandlung.
- Eine Reevaluation der Nierenersatztherapie muss alle 24 h erfolgen und ist im Dialyse- oder Hämofiltrationsprotokoll festzuhalten.
- Die Adäquatheit der applizierten Dosis der Nierenersatztherapie (NET-Dosis, „RRT delivered dose“) muss einmal täglich evaluiert werden und ist in einem Protokoll festzuhalten.
- Für kontinuierliche Verfahren ist als NET-Dosis die Effluatmenge festzuhalten (Substituat‑/Dialysat- plus Ultrafiltrationsvolumen) in ml/kg und Stunde. Es wird empfohlen, eine Dosis von 25–30 ml/kg und Stunde zu verschreiben, um Behandlungsunterbrechungen durch Filterwechsel, Clotting oder Transporte zu kompensieren und das Ziel von 20–25 ml/kg und Stunde zu erreichen.
- Für intermittierende Verfahren sind für eine NET-Dosis Behandlungsdauer, Blutfluss und Dialysatfluss zu dokumentieren. Eine kt/V-Messung ist für Patienten mit AKI nicht validiert.
- Die NET-Dosis sollte täglich reevaluiert und unter Bewertung von Serumparametern wie Kreatinin, Harnstoff, Phosphat und pH angepasst werden, um eine Unter- oder Überdialyse zu vermeiden.
- Nach Abschluss der Behandlung sollte jede AKI mit Stadium und ggf. durchgeführten Nierenersatztherapien im Entlassarztbrief aufgeführt werden. Dieser muss einen Hinweis enthalten, dass eine zeitnahe adäquate Nachsorge mit Kontrolle der Nierenfunktion durchgeführt werden sollte.

## Dokumentation und Ergebnisqualität

Die Sektion Niere der DIVI schlägt unter Berücksichtigung der spezifischen Versorgungsstruktur in Deutschland vor, folgende Daten zu erfassen:

- Gesamtzahl der mit Nierenersatztherapie behandelten Patienten;
- verwendete Modalitäten (Dialyse, Filtration, Hybridverfahren, Antikoagulation).

## Mindestmengen

Der Sektion Niere der DIVI ist bewusst, dass derzeit unter dem Aspekt der Qualitätssicherung in verschiedenen Bereichen der Krankenhausmedizin eine Diskussion um zu erbringende Mindestmengen geführt wird. Damit für eine Leistung eine Mindestmenge beschlossen werden kann, müssen laut G‑BA 2 Voraussetzungen erfüllt werden:

- die Leistung muss planbar sein;
- die Qualität des Behandlungsergebnisses muss von der Menge der erbrachten Leistungen abhängen.

Hinsichtlich der Planbarkeit der Leistung kann für eine Versorgungseinheit allenfalls ein statistischer Durchschnitt der in den vergangenen Jahren erbrachten Leistung ermittelt werden. Veränderungen in der Kapazität einer Intensivstation oder auch im zu versorgenden Patientenspektrum können zu starken Schwankungen in der Inzidenz einer AKI führen. Die Leistung Nierenersatztherapie als Notfallbehandlung ist daher nur schwer planbar.

Es erscheint plausibel, dass eine Beziehung zwischen der Menge der erbrachten Leistung und der Qualität des Behandlungsergebnisses für die Nierenersatztherapie besteht. Allerdings ist bisher in keiner wissenschaftlichen Studie eine direkte Korrelation nachgewiesen worden. Eine evidenzbasierte Festlegung von Mindestmengen ist daher derzeit nicht möglich. Darüber hinaus sind weitere Fragen offen, z. B. ob eine Gesamtmindestmenge für jedes einzelne Nierenersatzverfahren zu definieren ist oder ob alle Formen in einer Mindestmenge zusammengefasst werden sollen. Schließlich besteht nach Kenntnisstand der Sektion Niere der DIVI derzeit in keinem europäischen Land eine solche Mindestmengenregelung für die Durchführung der Nierenersatztherapie. Die Frage einer Mindestmengenregelung sollte daher zunächst zurückgestellt werden und nach Etablierung der o. g. Struktur- und Prozessvoraussetzungen zu einem angemessenen Zeitpunkt erneut evaluiert werden.

## Fazit

Die Behandlung von kritisch kranken Patienten mit schwerer AKI und Nierenersatztherapie erfordert spezielle Kenntnisse, etablierte Strukturen und definierte Prozesse, um eine hohe Behandlungsqualität in einem komplexen Umfeld zu gewährleisten.

*Strukturell* sollten neben technischen Voraussetzungen (Sonographie, Labor, Urindiagnostik) eine intensivmedizinische Expertise entsprechend den Anforderungen der intensivmedizinischen Komplexbehandlung, regelmäßige interprofessionelle Fortbildungen sowie die Verfügbarkeit eines nephrologischen und urologischen Konsils garantiert sein.

*Prozedural* sollten alle relevanten Aspekte der Behandlung dieser Patienten durch standardisierte Verfahrensanweisungen geregelt sein.
